# Inhibitory activities of selected Kampo formulations on human aldose reductase

**DOI:** 10.1186/1472-6882-14-435

**Published:** 2014-11-06

**Authors:** Toshihisa Onoda, Chikako Ishikawa, Takahiro Fukazawa, Wei Li, Masahiko Obayashi, Kazuo Koike

**Affiliations:** Faculty of Pharmaceutical Sciences, Toho University, Miyama 2-2-1, Funabashi, Chiba, 274-8510 Japan; Toho University Sakura Medical Center, Shimoshidu 564-1, Sakura, Chiba, 285-8741 Japan

**Keywords:** Kampo, Aldose reductase, Diabetes complications, Chotosan

## Abstract

**Background:**

Diabetes complications include various symptoms such as diabetic neuropathy and cognitive disorders. Aldose reductase (AR) is the rate-limiting enzyme of the polyol pathway and is one of the causal factors of diabetes complications. In this study, the bioactivities of eight selected Kampo formulations that are currently in clinical use for diabetes complications were assessed using human AR (hAR) inhibitory activity as the primary parameter to explore the possibilities of novel clinical applications of these formulations in the treatment of diabetes complications.

**Methods:**

The hAR inhibitory activities of four Kampo formulations that are clinically used for diabetic neuropathy, four Kampo formulations that are used for cognitive disorders, and a total of 21 component crude drugs were measured. Furthermore, the hAR inhibitory activity of Glycyrrhizae Radix preparata was measured to determine the effect of frying, which is one of the specific processing of Glycyrrhizae Radix. hAR inhibitory activity was determined by measuring the rate of decline in the absorbance of NAPH at 340 nm using 0.5 mM NADPH, 10 mM D,L-glyceraldehyde, and 3.6 mU/mL hAR in phosphate buffer solution (0.2 M, pH 6.2).

**Results:**

All of the Kampo formulations exhibited significant hAR inhibitory activity; Chotosan exhibited particularly strong activity. Among the 21 crude drugs tested, adequate inhibitory activities were found for the following, in descending order of activity: Glycyrrhizae Radix > Paeoniae Radix > Chrysanthemi Flos > Cinnamomi Cortex > Phellodendri Cortex > Uncariae Uncis cum Ramulus > Bupleuri Radix. Glycyrrhizae Radix preparata exhibited an inhibitory activity that was nearly identical to that of Glycyrrhizae Radix.

**Conclusions:**

Despite their seemingly different treatment objectives, all of the Kampo formulations that are clinically used for diabetes complications demonstrated significant hAR inhibitory activity. This activity might underlie the characteristic multi-target effects of Kampo formulations. Although the overall effect of a Kampo formulation is certainly difficult to evaluate based on specific herbal medications or components, the approach as taken in this study might nonetheless contribute to further advancement in the development of new drugs via the review of proper usage and re-examination of the chemical compounds from a new perspective.

## Background

Diabetes is primarily characterized by chronic hyperglycemia, and its worldwide prevalence is increasing [1]. The main purpose of diabetes control is the preventions of complications and the hindrance of disease progression. Diabetes complications are broadly divided into acute and chronic complications. Specifically, chronic complications involve major problems, such as diabetic neuropathy (DN), diabetic nephropathy, and diabetic retinopathy, that are caused by vascular disorders [2] and have been reported to be important risk factors for cognitive disorders [3, 4].

The pathogeneses of diabetes complications are mediated by a broad array of factors, such as the formation of vascular lesions via the reconstruction of vascular walls due to the formation of advanced glycation end products (AGE) that are associated with the accelerated nonenzymatic protein glycosylation (glycation), enhancement of oxidative stress due to active enzyme generation following glycation, blood flow disorders and neovascularization via PKC-β2 activation in vascular smooth muscle and endothelial cells, and enhanced metabolism in the polyol pathway via aldose reductase (AR) [5–8]. The polyol pathway is one of alternative pathways of the glycolytic pathway and contains the rate-limiting enzyme AR and sorbitol dehydrogenase (SDH). Because a large amount of glucose flows into the peripheral nerve cells in diabetic conditions, the excess glucose flows into the polyol pathway and is converted to sorbitol by AR. Sorbitol accumulates in cells and causes increases in osmotic pressure that lead to the suppression of cell functions and eventually peripheral neuropathy [8]. Additionally, depletion of the coenzyme NADPH causes the suppression of nitric oxide synthase activity, reduction of reduced glutathione production, and aberrant activation of PKC, which lead to the onset of peripheral neuropathy [9].

While blood sugar control is the basic treatment for diabetes complications, symptomatic treatments are also necessary. Among the latter treatments, vascular endothelial growth factor (VEGF) has been shown to be involved in diabetic macular edema, and intravitrial infusion of the VEGF inhibitor bevacizumab has been receiving increasing attention in recent years [10]. Losartan potassium is an angiotensin II AT_1_ receptor antagonist that is used clinically as an anti-hypertensive drug and has been approved for diabetic nephropathy [11]. The inhibition of AR, which is the rate-limiting enzyme of the polyol pathway, is considered effective for the treatment of DN, and epalrestat is the only oral drug that is currently approved for such use Japan [12]. However, epalrestat is associated with adverse effects, such as hepatic dysfunction, nausea, and gastrointestinal disorders such as diarrhea, which lower quality-of-life (QOL) and make the long-term use of this medication difficult [13]. Thus, there is a constant demand for novel drugs for the treatment of diabetes complications.

Although Kampo formulations are clinically used in various disease areas, and such use is primarily based on classic clinical theories, evidence-based clinical applications have begun to be established in recent years through the elucidation of the mechanisms of action of formulations such as Daikenchuto [14]. Kampo formulations are composed of combinations of various herbal medications and typically exert therapeutic effects through the integration of multi-component, multi-mode actions. Diabetes complications are linked to various diseases and mechanisms, which make Kampo formulations ideal as potential therapeutics. For example, Goshajinkigan is clinically used for DN [15], Keishibukuryogan is expected to be effective against diabetic nephropathy [16], and Chotosan and Yokukansan are used for the cognitive disorder and associated peripheral symptoms that are common among diabetic patients [17, 18].

Because AR reduces glucose in its role as an important enzyme in the polyol pathway, previous studies have reported that the inhibition of AR is involved in the action of Kampo medicines that are used for treatment of DN including Goshajinkigan, Hachimijiogan, Sokeikakketuto and Keishikajutsubuto [19]. In the present study, the bioactivities of eight selected Kampo formulations that are currently in clinical use for the treatment of diabetes complications were assessed using human AR (hAR) inhibitory activity as the primary parameter to explore their possible novel clinical applications in the treatment of diabetes complications. To identify the herbal medications that were responsible for the major hAR inhibitory activities, the component crude drugs were also examined.

## Methods

### Chemical reagents

All crude drugs and the Glycyrrhizae Radix preparata used in this study were purchased from Tochimoto Tenkaido Co., Ltd. (Osaka, Japan). Aldose reductase (EC1.1.1.21, 1-316aa, human, recombinant, E coli) was purchased from ATGen Co., Ltd. (Korea). D,L-glyceraldehyde and dimethyl sulfoxide (special grade) were purchased from Wako Pure Chemicals Industries, Ltd. (Osaka, Japan), and β-NADPH was purchased from Oriental Yeast Co. (Tokyo, Japan). Epalrestat was synthesized by Dr. Ryota Saito (Faculty of Science, Toho University).

### Manufacture of Kampo medicines and herbal medicines

The Kampo formulations at doses that were equivalent to those stipulated in the Japanese Pharmacopoeia and the crude drugs at 60 g each were infused in 600 mL of purified water for 1 h. The infusion was filtered, frozen in a freezer, and then lyophilized. The lyophilizate was used as the sample for the AR inhibitory activity assay. Table 1 shows the distributions of the constitution crude drugs in the eight Kampo formulations.

**Table 1:** **Crude drug constituents of eight Kampo formulations**

Crude drug	Kampo formulation ^a)^							
	Chotosan	Gosha jinkigan	Hachimi jiogan	Keishika jutsubuto	Shichimotsu kokato	Sokei- kakketuto	Yokukansan	Yokukansanka chinpihange
Glycyrrhizae Radix	1			1		1.0	1.5	1.5
*Paeoniae Radix*				2	4	2.5		
*Chrysanthemi Flos*	2							
*Cinnamomi Cortex*		1	1	2				
*Phellodendri Cortex*					2			
*Uncariae Uncis cum Ramulus*	3				3		3	3
*Bupleuri Radix*							2	2
*Cnidii Rhizoma*					3	2	3	3
*Atractylodis Lanceae Rhizoma*				2		2	4	4
*Plantaginis Semen*		3						
*Angelicae Radix*					4	2	3	3
*Astragali Radix*					3			
*Saposhnikoviae Radix*	2					1.5		
*Aurantii Nobilis Pericarpium*	3					1.5		3
*Zingiberis Rhizoma*	1			0.5		0.5		
*Ophiopogonis Tuber*	3							
*Ginseng Radix*	2							
*Poria Sclerotium*	3	3	3			2	4	4
*Rehmanniae Radix*		5	5		3	2		
*Pinelliae Tuber*	3							5
*Gypsum Fibrosum*	5							
*Achyranthis Radix*		3				1.5		
*Aconiti Tuber*		1	1	0.25				
*Alismatis Rhizoma*		3	3					
*Angelicae Dahuricae Radix*						1.0		
*Clematidis Radix*						1.5		
*Corni Fructus*		3	3					
*Batatatis Rhizoma*		3	3					
*Gentianae Scabrae Radix*						1.5		
*Zizyphi Fructus*				2				
*Moutan Cortex*		3	3					
*Notopterygii Rhizoma*						1.5		
*Persicae Semen*						2		
*Sinomeni Caulis et Rhizoma*						1.5		

^a)^Composition ratios of the crude drugs in g.

### AR assay

The AR inhibitory activities were measured according to the method described by Hyun et al. [20] with modifications. Briefly, 1 mL of the enzyme reaction solution contained 0.5 mM NADPH, 10 mM D,L-glyceraldehyde, and 3.6 mU/mL hAR in phosphate buffer solution (0.2 M, pH 6.2). The reaction was initiated by adding the substrate D,L-glyceraldehyde and measured by recording the decrease in the absorbance of NADPH at 340 nm. The enzyme content in 1 unit of enzyme allowed for the analysis of the oxidation of 1 μmol NADPH/min at 25°C. Absorbance was measured using a Shimadzu UV-24 ultraviolet–visible spectrophotometer (Shimadzu Co., Kyoto, Japan). In this study, the known AR inhibitor epalrestat was used as the positive control.

### Inhibition rate and IC_50_

The AR inhibitory activity of each sample was determined by measuring the change in NADPH absorbance at 340 nm. The inhibition rate was calculated using the following formula:


where Δ*A*_sample_, Δ*A*_control_ and Δ*A*_blank_ denote changes in absorbance at 340 nm in the presence of sample, in the absence of sample, and in the absence of enzyme, respectively. Δ_control_ represents the normal progress of the reducing reaction in the absence of sample and was used to define 0% inhibition. Δ_blank_ represents the spontaneous degradation of NADPH and was used to define 100% inhibition. All samples were assayed in the concentration range of 1 μg/mL to 100 μg/mL. The IC_50_ values were determined by linear regression analyses.

### Unit definition

Kampo formulations and epalrestat are currently used clinically. Therefore, their inhibitory potencies could be compared based on actual doses. The hAR inhibition per daily dose was defined as 1 unit as indicated on the package insert.

## Result and discussion

### hAR inhibitory activities of the Kampo formulations

The *in vitro* hAR inhibitory activities were measured to evaluate the potential actions of the eight selected Kampo formulations. Goshajinkigan, Sokeikakketuto, Keishikajutsubuto and Hachimijiogan are clinically used for the treatment of peripheral neuropathy and were used as controls because previous reports exist only for these four formulations [19]. Four Kampo formulations, namely, Chotosan, Shichimotsukokato, Yokukansan, Yokukansankachinpihange, which are used for cognitive disorders were also selected to determine their AR inhibitory activities in this study (Table 2). The inhibitory activities of the samples were measured at concentrations that ranged from 20 μg/mL to 100 μg/mL, and the IC_50_ values were determined using linear regression to compare the inhibition potencies (Table 2). All of the tested Kampo formulations exhibited significant inhibitory activities, and the most potent of the eight formulations was Chotosan (IC_50_: 43.6 μg/mL).

**Table 2:** **hAR inhibitory activities of the Kampo formulations**

Kampo formulation	IC _50_^a)^	
	μg/mL	μUnit/mL
Chotosan	43.6 (±3.1)	5.81
Yokukansan	48.9 (±6.8)	6.52
Keishikajutsubuto	50.2 (±3.5)	6.69
Goshajinkigan	56.7 (±22.3)	7.56
Sokeikakketuto	68.4 (±9.2)	9.12
Shichimotsukokato	69.2 (±11.1)	9.23
Hachimijiogan	71.6 (±44.0)	9.55
Yokukansankachinpihange	83.4 (±10.4)	11.12
epalrestat ^b)^	0.014 (±0.003)	0.098

^a)^The values are presented as the means (± SD), *n* = 3.

^b)^Positive control.

The group of Kampo formulations that are clinically used for DN and for which AR inhibitory activities in the rat lens have been reported [19] exhibited significant hAR inhibiting effects in the present *in vitro* study. Moreover, Chotosan and Yokukansan exhibited greater AR inhibitory activities than this group of Kampo formulations. Because there are reports that Goshajinkigan is effective in the treatment of diabetes complications and exhibits AR inhibition [15, 19], Chotosan and Yokukansan might also be effective for diabetes complications based on the AR-inhibiting activities. Further studies and clinical reports are expected to demonstrate this conjecture in the future.

Chotosan is also used for the treatment of hypertension and has a protective effect on the endothelium [21]. Because AR inhibitors have been reported to negate diabetes-evoked hypertension via the amelioration of impaired endothelial relaxation and NO production [22], the AR inhibitory activity of Chotosan might contribute to the mechanisms of its anti-hypertensive effect.

### Comparison to epalrestat

Because epalrestat is actually used clinically, it was used as the positive control in this study. The levels of hAR inhibition of the individual drugs were compared based on actual doses. As shown in Table 1, epalrestat exhibited much greater hAR inhibitory activity than did any of the Kampo formulations when their IC_50_ values were expressed in μUnit/mL. However, epalrestat is associated with the side effect of severe liver damage and is difficult to use [13]. Although the Kampo formulations exhibited weaker AR inhibitory activities than did epalrestat, the potential *in vivo* effects of these formulations can only be extrapolated based on these *in vitro* results. Additionally, the reduced side effects and multi-function properties of these Kampo formulations can be exploited. Because epalrestat has been used in combination with mecobalamin or mexiletine in clinical practice, combination therapies might also be favorable for the clinical application of these Kampo formulations, but further evidence is required to support this supposition.

### Evaluations of the crude drugs

Because the Kampo formulations were found to exhibit significant inhibitory activities in the present study, a total of 21 crude drugs were further assessed for hAR inhibitory activity to identify their contributions to overall hAR inhibitory activities of the Kampo formulations. Because examinations of the crude drugs have been performed in the past [19], we focused on the crude drugs of the Kampo formulations, including *Uncariae Uncis cum Ramulus,* in the present study. The inhibitory activities of the crude drug samples was measured over a concentration range from 1 μg/mL to 100 μg/mL, and the IC_50_ values of 50 μg/mL and lower were considered to be indicative of significant inhibitory activity (Table 3). Of the 21 crude drugs that were assayed, adequate inhibitory activities were observed for the following drugs (in descending order of activity): Glycyrrhizae Radix, Paeoniae Radix, Chrysanthemi Flos, Cinnamomi Cortex, Phellodendri Cortex, Uncariae Uncis cum Ramulus, and Bupleuri Radix.

**Table 3:** **The hAR inhibitory activities of the crude drugs**

Crude drug	IC _50_^a)^
*Glycyrrhizae Radix*	7.6 (±2.4)
*Paeoniae Radix*	14.2 (±0.3)
*Chrysanthemi Flos*	22.5 (±1.6)
*Cinnamomi Cortex*	26.8 (±9.0)
*Phellodendri Cortex*	32.1 (±5.8)
*Uncariae Uncis cum Ramulus*	36.4 (±0.1)
*Bupleuri Radix*	47.5 (±8.5)
*Cnidii Rhizoma*	> 50
*Atractylodis Lanceae Rhizoma*	> 50
*Plantaginis Semen*	> 50
*Angelicae Radix*	> 50
*Astragali Radix*	> 50
*Saposhnikoviae Radix*	> 50
*Aurantii Nobilis Pericarpium*	> 50
*Zingiberis Rhizoma*	> 50
*Ophiopogonis Tuber*	> 50
*Ginseng Radix*	> 50
*Poria Sclerotium*	> 50
*Rehmanniae Radix*	> 50
*Pinelliae Tuber*	> 50
*Gypsum Fibrosum*	> 50

^a)^The values are presented as the means (± SD), n = 3, IC_50_ in μg/mL.

The results revealed that Glycyrrhizae Radix, Paeoniae Radix, and Cinnamomi Cortex exhibited significant hAR inhibitory activities, and Atractylodis Lanceae Rhizoma and Zingiberis Rhizoma exhibited weak hAR inhibitory activities; these findings are consistent with those of previous report that examined the inhibitory activities of drugs against AR from rat lenses [19]. The hAR inhibitory activities of Phellodendri Cortex, Uncariae Uncis cum Ramulus, and Bupleuri Radix are novel findings.

Multiple components have been suggested to be responsible for the hAR inhibitory activities of the clinically used drugs Goshajinkigan and Chotosan, which exhibited strong hAR inhibitory activities in this study. This suggestion is based on the fact that Poria Sclerotium is the only common crude drug between the two Kampo formulations, and Poria Sclerotium alone did not exhibit a detectable hAR inhibitory activity. The hAR inhibitory activity of Chotosan might be primarily attributable to Glycyrrhizae Radix, which has been reported to have an AR-inhibiting effect [23], or to Uncariae Uncis cum Ramulus or Chrysanthemi Flos, which were observed to possess hAR inhibitory activities the present study and are thought to ameliorate the numbness and headache that occur due to strong vital substance deficiencies (a state that is devoid of vigor and drive).

### Influence in the specific processing

We investigated whether the hAR inhibitory activities vary across the traditional methods herbal medications usage. In the present study, we focused on Glycyrrhizae Radix preparata, because it is sometimes used in Kampo formulations with specific processing, but other constituent crude drugs in this study are not. The frying of Glycyrrhizae Radix is a traditional method that is used to enhance its vital substance tonification effect, but there are few reports regarding the differences between Glycyrrhizae Radix and Glycyrrhizae Radix preparata. The effect of frying on hAR inhibitory activity was investigated in the present study via measurements of the hAR inhibitory activity of Glycyrrhizae Radix preparata. Glycyrrhizae Radix preparata and Glycyrrhizae Radix exhibited inhibitory activities that were nearly equivalent (IC_50_s: 7.5 μg/mL and 7.6 μg/mL, respectively). Although the changes in the chemical constituents that occur due to frying are unknown, it is likely that frying has no effect on the hAR inhibitory activity of Glycyrrhizae Radix.

### Consideration of the ingredients

There have been several previous reports related to the specific chemical components of herbal medications with strong hAR inhibitory activities. Isoliquiritigenin is an analgesic and spasmolytic component of Glycyrrhizae Radix that has received particular attention and has been reported to exhibit AR inhibitory activity [23]. One study found that isoliquiritigenin was the ingredient of Glycyrrhiza Radix that exhibited the greatest AR inhibitory activity among the crude drugs that were measured in that study; moreover, Yokukansan also contains Isoliquiritigenin [24]. Additionally, Isoliquiritigenin has been reported to also exhibit advantageous effects against diabetes complications that include antiplatelet [24] and anti-oxidative effects [25]. Based on these past reports that found that isoliquiritigenin exhibits AR inhibitory activity and is contained in Yokukansan, further research into this AR inhibiting compound can be expected in the future.

Glycyrrhizae Radix is contained in Yokukansankachinpihange, which was found to exhibit the weakest activity among the eight Kampo formulations that were tested in this study. Kampo formulations often have multiple active components and chemical compounds and multiple target sites, and synergistic actions of these multiple ingredients are often present. Therefore, it is difficult to fully clarify the detailed effects and mechanisms of these formulations, which might exhibit complex characteristics that extent beyond the sums of the activities of each of the crude drugs in isolation.

## Conclusions

All of the Kampo formulations that are clinically used for diabetes complications exhibited high hAR inhibitory activities despite the seemingly different treatment objectives of the uses of these formulations. These findings might be attributable to the multi-target effects of the Kampo formulations. Although the overall effect of a Kampo formulation is certainly difficult to evaluate based its specific herbal medications and components, the identification of Kampo formulations with strong activity on a specific target, subsequent examination of the constituent crude drugs of those formulations, and discussion of the chemical components might nonetheless contribute to further advancements in the development of new drugs based on review of the proper use of the formulation and re-examination of the chemical compounds from a new perspective.
